# Targeted Chiral Analysis of Bioactive Arachidonic Acid Metabolites Using Liquid-Chromatography-Mass Spectrometry

**DOI:** 10.3390/metabo2020337

**Published:** 2012-04-20

**Authors:** Clementina Mesaros, Ian A. Blair

**Affiliations:** Centers for Cancer Pharmacology and Excellence in Environmental Toxicology, Department of Pharmacology, University of Pennsylvania School of Medicine, Philadelphia, PA 19104, USA; Email: mesaros@ mail.med.upen.edu (C.M.)

**Keywords:** arachidonic acid, eicosanoids, chiral liquid chromatography, tandem mass spectrometry, selected reaction monitoring, atmospheric pressure chemical ionization, electron capture, cyclooxygenase, lipoxygenase, cytochrome P-450

## Abstract

A complex structurally diverse series of eicosanoids arises from the metabolism of arachidonic acid. The metabolic profile is further complicated by the enantioselectivity of eicosanoid formation and the variety of regioisomers that arise. In order to investigate the metabolism of arachidonic acid *in vitro* or *in vivo*, targeted methods are advantageous in order to distinguish between the complex isomeric mixtures that can arise by different metabolic pathways. Over the last several years this targeted approach has become more popular, although there are still relatively few examples where chiral targeted approaches have been employed to directly analyze complex enantiomeric mixtures. To efficiently conduct targeted eicosanoid analyses, LC separations are coupled with collision induced dissociation (CID) and tandem mass spectrometry (MS/MS). Product ion profiles are often diagnostic for particular regioisomers. The highest sensitivity that can be achieved involves the use of selected reaction monitoring/mass spectrometry (SRM/MS); whereas the highest specificity is obtained with an SRM transitions between an intense parent ion, which contains the intact molecule (M) and a structurally significant product ion. This review article provides an overview of arachidonic acid metabolism and targeted chiral methods that have been utilized for the analysis of the structurally diverse eicosanoids that arise.

## 1. Introduction

**Figure 1:**
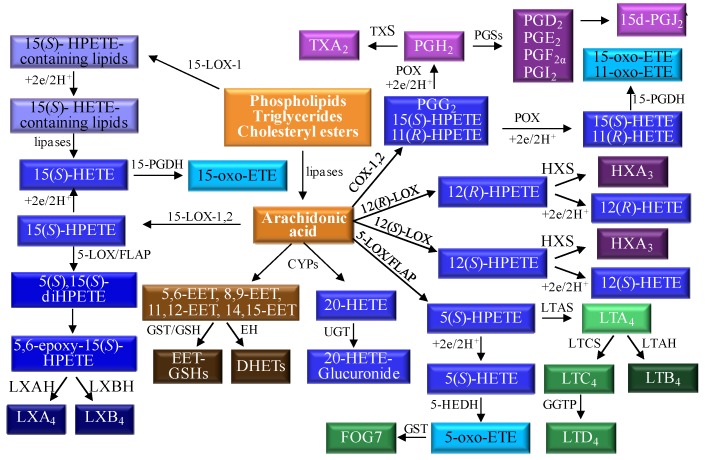
Pathways of arachidonic acid metabolism. Abbreviations: COX, cyclooxygenase; CYP, cytochrome P540; EET, epoxyeicosatrienoic acid; EH, epoxide hydrolase; FLAP, 5-lipoxygenase activating protein; GGT, γ-glutamyltranspeptidase; GSH, glutathione; GST, glutathione-*S*-transferase; H, hydrolase; HEDH, hydroxyeicosanoid dehydrogenase; HETE, hydroxyeicosatetraenoic acid; HPETE, hydroperoxyeicosatetraenoic acid; HX, hepoxillin; LOX, lipoxygenase; LT, leukotriene; LX, lipoxins; PG, prostaglandin; PGDH, prostaglandin dehydrogenase; POX, peroxidase; S, synthase; TX thromboxane; UGT, UDP-glucuronosyltransferases.

Arachidonic acid is metabolized to an array of oxidized bioactive lipids by a series of different oxygenases that can introduce molecular oxygen with extraordinary regioselectivity and stereospecificity (**Figure 1**). Free arachidonic acid serves as the substrate for cyclooxygenases (COXs), lipoxygenases (LOXs), and cytochromes P-450 (CYPs); whereas esterified arachidonic acid is primarily metabolized by 15-LOX-1. The ability of COXs to convert arachidonic acid to prostaglandins (PGs) and thromboxane A_2_ was recognized over 50 years ago [1,2,3]. Two COX isoforms have been identified, the first of which, COX-1, is constitutively active [4]. The presence of a second inducible form of COX was first suggested by experiments, which showed a transient increase in the formation of PGE_2_ from arachidonic acid by canine kidney cells upon stimulation with tumor promoters and carcinogens [5,6]. The increased PGE_2_ production was eliminated by inhibition of transcription or translation, indicating that it was dependent upon *de novo* COX synthesis. This new isoform (COX-2) was subsequently cloned, sequenced, and its expression was found to be inducible in human cells [7]. COX-2 and COX-1 share 60% sequence homology [8] and they are both responsible for the metabolism of free arachidonic acid to the bioactive PGs and TXA_2_ (**Figure 1**).

Arachidonic acid is converted initially to the hydroperoxy-endoperoxide PGG_2_, which subsequently converts to the hydroxy-endoperoxide PGH_2_ through the enzyme’s peroxidase (POX) activity (**Figure 1**) [9]. A variety of bioactive arachidonic acid metabolites are produced from PGH_2_, varying in function from regulating inflammation, blood clotting, ovulation, initiation of labor, bone metabolism, nerve growth and development, kidney function, and blood vessel tone. As such, changes to COX-2 expression help to regulate diverse functions in several tissues. It is not surprising then that alterations in normal COX-2 activity are seen in a number of diseases, ranging from cardiovascular disease to cancer [3]. Initial reports indicated that there was elevated COX-2 expression in colorectal cancer [10], and further studies showed that numerous other epithelial cancers were also associated with elevated COX-2 expression [11,12,13]. The presence of increased COX-2 activity in cancer appears to be associated with more aggressive phenotype [14,15]. For example, breast cancers with increased COX-2 expression had an increased rate of recurrence, increased metastasis, and worse clinical prognosis and survival rate [16,17]. Many of these adverse effects have been ascribed to increased formation of pro-proliferative COX-2-derived PGE_2_ [18]. More recently, it has been recognized that COX-mediated formation of 11(*R*)- and 15(*S*)-hydroperoxyeicosatetraenoic acid (HPETEs) followed by POX-mediated reduction to the corresponding 11(*R*)- and 15(*S*)-hydroxyeicosatetraenoic acids (HETEs) provides excellent substrates for 15-hydroxyprostaglandin dehydrogenase (15-PGDH) [19,20]. The resulting 11- and 15-oxo-eicosatetraenoic acids (ETEs) have anti-proliferative activity similar to that observed for 15-deoxy-Δ^12,14^-PGJ_2_ (15d-PGJ_2_) [21]. It is noteworthy that 15-PGDH is down-regulated in many cancers [22], which results in increased activity of pro-proliferative PGE_2_ (through decreased inactivation) and decreased activity of anti-proliferative 11- and 15-oxo-ETE (through decreased biosynthesis) [20].

The 5-LOX enzyme has a nuclear localization similar to the COXs and it is also able to efficiently metabolize arachidonic acid. 5-LOX-derived 5(*S*)-HPETE, is either reduced to 5(*S*)-HETE, or serves as a precursor to the formation of leukotrienes (LTs) B_4_, C_4_, and D_4_ (**Figure 1**) [23]. The formation of 5(*S*)-HPETE is critically dependent upon the presence of 5-lipoxygenase activating protein (FLAP) [24]. 5-LOX and FLAP are expressed primarily in inflammatory cells such as polymorphonuclear leukocytes, monocytes, macrophages, and mast cells [23,25,26,27]. Therefore, 5-LOX-mediated LT formation is thought to play a critical role in inflammation, and allergic disorders [28,29,30,31]. In addition, a number of studies have implicated 5-LOX-derived arachidonic acid metabolites as mediators of atherogenesis and heart disease [23,25,32]. The 5-LOX pathway of arachidonic acid metabolism has also been proposed to play a role in prostate and pancreatic cancer [33,34,35,36]. It is noteworthy that 5-HETE is efficiently converted to 5-oxo-ETE by 5-hydroxyeicosanoid dehydrogenase (5-HEDH) [37] analogous to the 15-PGDH-mediated conversion of 11(*R*)-HETE to 11-oxo-ETE [20] (**Figure 1**). The biosynthesis of 5-oxo-ETE is regulated by intracellular NADP^+^ levels and is increased under conditions that favor oxidation of NADPH to NADP^+^. This occurs, for example, during oxidative stress and during activation of the respiratory burst in phagocytic cells. 5-Oxo-ETE is a potent chemoattractant for eosinophils, neutrophils, basophils and monocytes, an activity that is thought to be mediated activation of the G_i/o_ coupled OXE receptor [38]. Glutathione-*S*-transferase (GST)-mediated metabolism of 5-oxo-ETE results in the formation of 5-oxo-7-glutathionyl-8,11,14-eicosatrienoic acid (FOG7), which has similar biological activity to the parent 5-oxo-ETE [39].

In contrast to 5-LOX, which strongly prefers free arachidonic acid as its substrate [40], mammalian 15-LOXs are capable of oxygenating both free and esterified polyunsaturated fatty acids [41]. 15-LOX can also oxidize more complex lipid-protein assemblies such as biomembranes and lipoproteins [42,43]. Type 1 human 15-LOX (15-LOX-1), which is mainly expressed by reticulocytes, eosinophils and macrophages, converts esterified arachidonic acid to esterified 15(*S*)-HPETE and a small amount of 12(*S*)-HPETE; [44]. 15-LOX-1 is a cytoplasmic enzyme with up-regulated expression in atherosclerotic lesions and at sites of macrophage accumulation [45]. Studies of 15-LOX-1 in hematopoietic cells have demonstrated that it translocates to the inner plasma membrane and other non-nuclear membranes (e.g. sub-mitochondrial membranes) after stimulation with calcium [46].

It has been suggested that 15-LOX-1 plays an important role in angiogenesis and carcinogenesis [47]. This stems from the observation that both angiogenesis and tumor formation in xenograft models were inhibited in transgenic mice that over-expressed 15-LOX-1 in their endothelial cells [48]. In contrast, 15-LOX has been shown to have both pro-inflammatory and anti-inflammatory effects in cell culture and primary cells and opposite effects on atherosclerosis in animal models [49]. Furthermore, there is substantial evidence that 15-LOX-1 has a pro-atherogenic effect including its direct contribution to LDL oxidation and to the recruitment of monocytes to the vessel wall [49]. The explanation for these conflicting observations might reside in the different biological effects of many lipid mediators generated by the 15-LOX-1 pathway. For example, 15-HETE is converted to 15-oxo-ETE, an anti-proliferative eicosanoid (**Figure 1**). Similarly, 5-LOX-mediated metabolism of 15-LOX-derived 15(*S*)-HPETE results in the formation of the anti-inflammatory lipoxins (LX) A_4_ and LXB_4_ [50] (**Figure 1**). Additional 15-LOX-1-derived lipid mediators arising from other polyunsaturated fatty acids such as eicosapentaenoic acid (E-resolvins) and docosahexaenoic acid (D-resolvins) could also potentially be involved [51].

A second human 15-LOX (15-LOX-2) was discovered by the Brash group in 1997, which in contrast to 15-LOX-1, does not efficiently metabolize linoleic acid [52]. 15-LOX-2 has a limited tissue distribution, with mRNA detected in prostate, lung, skin, and cornea, but not in numerous other tissues, including peripheral blood leukocytes [53]. However, a recent study has shown that significant expression of 15-LOX-2 occurs in tumor-associated macrophages [54]. The consequences of this finding are not yet fully understood.

12-LOX-mediated arachidonic acid metabolism results in the formation of 12(*S*)-HPETE through a human platelet-type 12(*S*)-LOX [55] or 12(*R*)-HPETE through a human skin-type 12(*R*)-LOX [56]. A 2electrons reduction of the HPETEs results in the formation of 12(*S*)-HETE or 12(*R*)-HETE, respectively. Although, 12(*S*)-HETE is a major product of platelet aggregation it is also found in high levels in tumors [57]. A specific orphan receptor for 12(*S*)-HETE was recently characterized [58]. Binding to the receptor was shown to result in activation of ERK1/2 MEK, and NF-κB as well as cell invasion, which suggested that this pathway could be involved in tumor metastases. In contrast 12(*R*)-HETE, plays a role in normal skin development and appears to be involved in the pathophysiology of psoriasis and other proliferative skin diseases [59]. Hepoxillin synthase converts the respective 12(*S*)- and 12(*R*)-HPETEs into hepoxillin A_3_ (HXA_3_) isomers, which are thought to be early mediators of inflammatory responses [60].

Cytochromes P450 (CYPs) are membrane bound hemoproteins that convert arachidonic acid into a series of oxidized lipid metabolites through three different pathways [61,62]. First, they can catalyze *bis*-allylic oxidation of to produce 7-, 10-, and 13-HETEs or lipoxygenase-like products such as 11-, 12-, and 15-HETEs [63]. Second, CYPs primarily of the 4 family, can perform conventional hydroxylation reactions on the ω-terminus of arachidonic acid to produce 16-, 17-, 18-, 19-, and 20-HETEs [64]. Interestingly, the 20-HETE resulting from ω-oxidation is excreted primarily as a glucuronide conjugate in human urine [65]. Third, CYPs can epoxidize arachidonic acid at each of the *cis*-olefins to produce four epoxyeicosatrienoic acid (EET) regioisomers (5,6-EET, 8,9-EET, 11,12-EET, 14,15-EET) (**Figure 1**) each of which can be formed as an enantiomeric pair [66,67,68]. The 5,6-EET regioisomer is rapidly converted to the corresponding lactone, due to the proximity of the terminal carboxylic group and the 5,6-epoxide [69]. However, the other EETs are relatively stable until they are metabolized either by cytosolic epoxide hydrolases (EHs) [70,71] to dihydroxyeicosatrienoic acids (DHETs) or by GSTs to form GSH-adducts [72]. The regioselectivity and enantioselectivity of EET formation is CYP-isoform specific and is thought to involve primarily CYPs from the 2 family in humans (2C8, 2C19, 2D6, and 2J2) [73,74,75]. Endogenous EETs [76,77,78], are normally re-esterified and are then found at the *sn-*2 position of cellular glycerophospholipids, so they can be readily released by basic hydrolysis [79,80]. The EETs have potent vasodilator [79,81,82] and anti-inflammatory activities [83,84,85,86]. In addition, depending upon their chirality and regiochemistry, the EETs can inhibit the platelet aggregation [73,87]. Finally, a recent study has shown that that elevated EETs have a potent stimulatory effect on primary tumor growth and tumor angiogenesis [88]. Furthermore, elevated EETs triggered massive, unprecedented patterns of metastatic spread and escape from tumor dormancy [88], raising concerns about therapeutic strategies that involve up-regulation of EETs [89]. 

## 2. Analysis of Arachidonic Acid-Derived Eicosanoids by Targeted LC-MS.

The profile of arachidonic acid metabolites is complicated by the enantioselectivity of eicosanoid formation as well as the variety of regioisomers that arise (**Figure 1**). In order to investigate the metabolism of arachidonic acid *in vitro* or *in vivo*, targeted chiral methods are advantageous, to help distinguish between the enantiomers that are formed by different pathways. In a 2009 review article [90], we observed that there were few reports of targeted approaches for more than one class of eicosanoids. Since that time, a number of targeted approaches have appeared [91,92,93,94,95,96,97,98,99,100] where more than one class of eicosanoid and/or other metabolites arising from the same metabolic pathway were analyzed [93]. To efficiently conduct targeted eicosanoid analyses, the LC separations are coupled with CID and MS/MS analysis. Product ion profiles are often diagnostic for particular regioisomers. The highest sensitivity that can be achieved for the analysis of eicosanoids involves the use of LC-SRM/MS. Highest specificity is obtained when the SRM transition is between an intense parent ion which contains the intact molecule (M) and a structurally significant product ion. An example of this useful situation arises with HETEs, where product ions are formed through α-cleavage adjacent to a double bond [101]. In some cases, fragment ions produced in the collision cell are not very specific, and isomeric eicosanoids sometimes produce very similar product ion profiles. An example of this less desirable situation arises with PGE_2_ and PGD_2_, where the isomers need to be well separated by LC for correct quantification. Most LC-SRM/MS methods that have been reported employ ESI in the negative ion mode, where the parent ion arises from de-protonation of the eicosanoid molecule (M) to give an ion corresponding to [M-H]^-^. LC has generally been performed using reversed stationary phases coupled with aqueous mobile phases [91,93,94,95]. Analyses are normally conducted using stable isotope dilution methodology with deuterium-labeled eicosanoid analogs as internal standards (ISTDs), which confer much greater specificity than structural analog ISTDs. Quantification is performed by constructing calibration curves for each analyte. Standard solutions of different concentrations are prepared by serial dilution from commercially available standard eicosanoids and they are spiked with the same amount of the deuterium labeled ISTD as the samples to be determined. However, most targeted methods do not use chiral chromatography and so they cannot distinguish between pairs of eicosanoid enantiomers.

**Figure 2:**
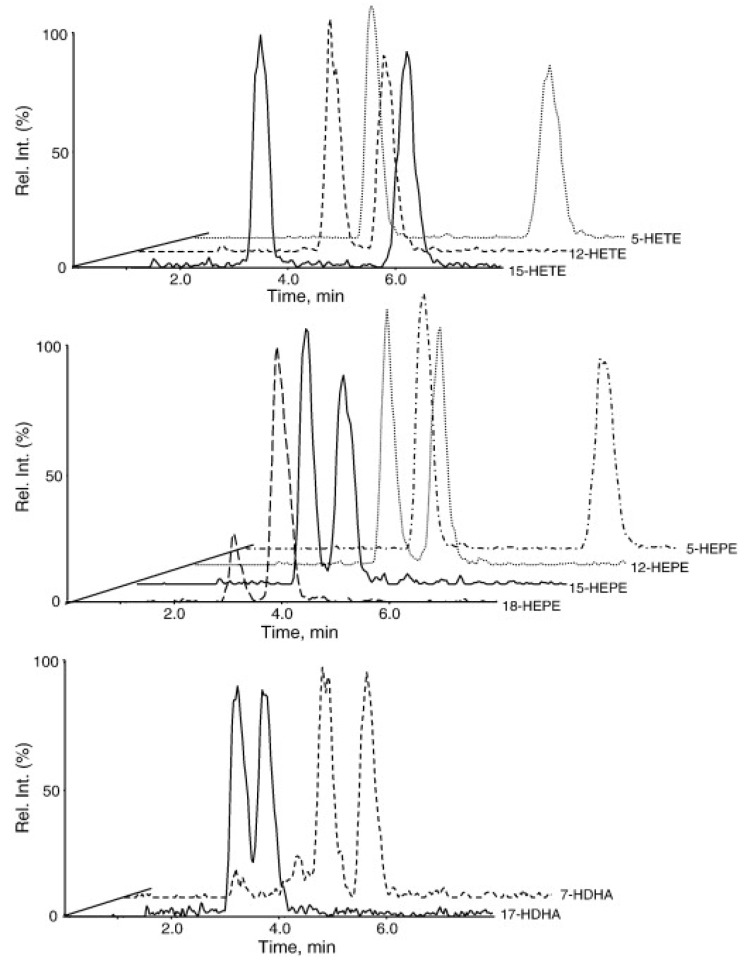
Chiral separation of HETEs (top panel) and hydroxylated metabolites of EPA (middle panel) and DHA (lower panel). Reprinted with permission from Ref. [93].

Electron capture atmospheric pressure chemical ionization (ECAPCI)-MS analysis of fatty acyls, which have been derivatized as pentafluorobenzyl (PFB) esters, is more sensitive when compared with ESI of un-derivatized fatty acyls [102]. This methodology can be readily coupled with chiral normal phase LC and so it enables chiral lipid peroxidation products to be resolved [103]. Chiral LC-ECAPCI/MS can be employed to determine whether the eicosanoids are derived from non-enzymatic or enzymatic pathways [104,105]. The low energy electrons generated in the APCI source (through interaction of the corona discharge with the nitrogen nebulizing gas) can be captured with a suitable electron-capturing group (such as PFB esters), similar to the process of electron capture negative chemical ionization (ECNCI), which occurs in a chemical ionization source during gas chromatography (GC-MS) analysis [106,107]. The initially formed radical anion dissociates (though dissociative electron capture) into an intense carboxylate anion, which is then subjected to CID and MS/MS analysis [103,105]. A recent targeted method developed by the Serhan group [93] is particularly appealing since it uses chiral reversed-phase (rather than normal phase) LC coupled with negative ESI. This method was able analyze the enantiomeric formation of 5, 12 and 15-HETEs, together with additional hydroxylated eicosanoids derived from eicosapentaenoic acid (EPA) and docosahexaenoic acid (DHA) (**Figure 2**).

**Table 1:** Chiral LC-MS separation for eicosanoids.

MS Method	Analytes	HPLCcolumn	Derivatization reagent	Starting mobile phase	References
ECAPCI	HETEs	Chiralpack AD-H	PFB-Br	Hexanes/isopropanol/Methanol (98:1:1)	[103,104,108,109]
ECAPCI	EETs	Chiralpack AD-H	PFB-Br	Hexanes/isopropanol(99.6:0.4)	[90]
ESI	5, 12 and 15-HETE	Chiralpack AD-RH	none	Methanol/water/acetic acid (95:5:0.1)	[93]

## 3. COX Mediated Metabolism

### 3.1. COX-2 Mediated Metabolism of Arachidonic Acid in Colorectal Adenocarcinoma Cells

**Figure 3:**
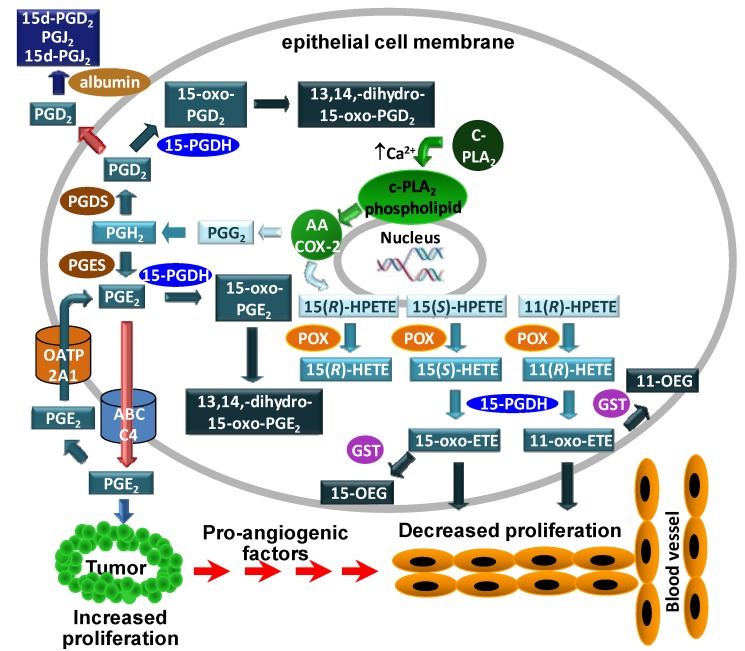
Formation and action of COX-2-derived eicosanoids in epithelial cell models. arachidonic acid is released from membrane phospholipids by calcium-dependent cytosolic phospholipase A_2_ (cPLA_2_). The released arachidonic acid undergoes COX-2-mediated metabolism to PGs or forms the lipid hydroperoxides, 15(*S*)-hydroperoxyeicosatetraenoic acid (HPETE), 15(*R*)-HPETE and 11(*R*)-HPETE, which are reduced to the corresponding HETEs. PGD_2_ and PGE_2_ are inactivated by 15-PGDH-mediated conversion to their 15-oxo metabolites. Both 15-oxo-PGD_2_ and 15-oxo-PGE_2_ are converted to 13,14-dihydro-5-oxo-PG metabolites. Intact PGD_2_ secreted by the epithelial cells can undergo albumin-mediated dehydration to 15d-PGJ_2_. PGE_2_ secreted from the epithelial cells by the ABCC4 transporter is pro-proliferative for tumor cells. Reuptake of PGE_2_ by OATP2A1 into the epithelial cells leads to further 15-PGDH-mediated inactivation. In contrast to PGE_2_ and PGD_2_, 15(*S*)-HETE and 11(*R*)-HETE are activated by 15-PGDH-mediated oxidation to 15-oxo-ETE and 11-oxo-ETE, respectively. The oxo-ETEs are further conjugated to form OEGs. Secreted 15- and 11-oxo-ETE that escape further metabolism can then inhibit endothelial cell proliferation. Therefore, down-regulation of 15-PGDH and OATP2A1 would result in increased PGE_2_-mediated tumor and endothelial cell proliferation. Reprinted with permission from Ref [110].

Using our targeted chiral lipidomics approach, the COX-2 metabolism of arachidonic acid in the epithelial cells showed that 11(*R*)-HETE is the primary hydroxylated metabolite produced [19], and the PGs were the most abundant metabolites. COX-2 expression is unregulated by different toxic molecules [111,112,113], and the products will in turn regulate other intracellular pathways. PGE_2_ is the main PG formed by COX-2 and it is further metabolized by 15-PGDH to the inactive form, 15-oxo-PGE_2_, which is further metabolized to 13,14-dihydro-15-oxo-PGE_2_. Increased PGE_2_ activity due the loss of 15-PGDH expression is implicated in tumor formation [22,114,115,116,117]. 15-PGDH also converts PGD_2_ into 15-oxo-PGD_2_ (**Figure 3**).

11(*R*)-HETE, 15(*S*)-HETE and 15(*R*)-HETE are also produced by COXs, from the corresponding hydroperoxides (**Figure 3**). It is well established that 15(*S*)-HETE is metabolized to 15-oxo-ETE [118,119]. Using chiral LC-ECAPCI/MS, 11-oxo-ETE formation by epithelial cells expressing COX-2 was investigated and the dehydrogenase responsible for the transformation was identified [110]. First, the biosynthesis of 11-oxo-ETE was conducted using11(*R*)-HETE and recombinant 15-PGDH [20]. The catalytic activity of 15-PGDH was approximately one-third that for 15(*S*)-HETE but nevertheless, it efficiently produced the corresponding 11-oxo-ETE. This result was quite unexpected, since 11(*R*)-HETE lacks the 15(*S*)-hydroxyl group that is normally required by the 15-PGDH enzyme. The identity of the newly formed 11-oxo-ETE was established by comparison with an authentic standard. The product of the 15-PGDH catalyzed reaction of 11(*R*)-HETE had the same MS/MS spectrum as an authentic standard and the same chromatographic properties [20].

**Figure 4:**
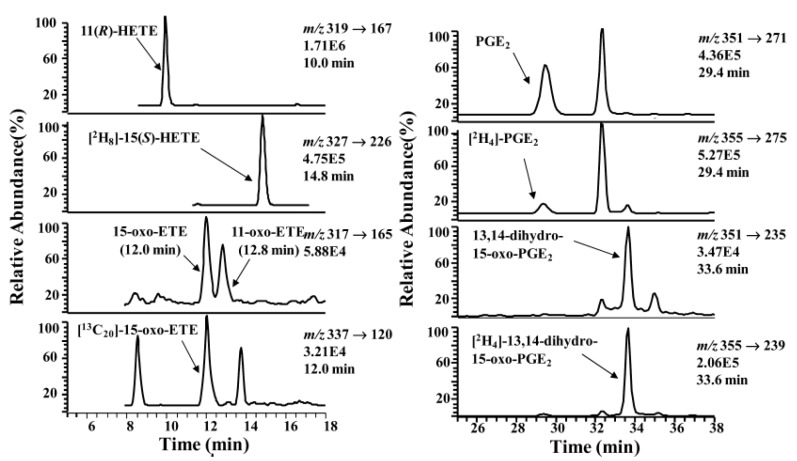
Targeted chiral lipidomics analysis of COX-2-derived eicosanoids from LoVo cells. LoVo cells were lysed; eicosanoids were extracted, derivatized with PFB bromide, and analyzed by LC-ECAPCI/SRM/MS. LoVo cell lysates were pretreated with 50 μM CAY10397 to inhibit 15-PGDH to be able to detect the 11-, 15-HETEs and PGE_2_. Representative chromatograms are shown for (top to bottom) (a) 11(*R*)-HETE-PFB (*m*/*z* 319 → 167), (b) [^2^H_8_]-15(*S*)-HETE-PFB internal standard (*m*/*z* 327 → 226), (c) 11-oxo-ETE-PFB (*m*/*z* 317 → 165) and 15-oxo-ETE-PFB (*m*/*z* 317 → 165), (d) [^13^C_20_]-15-oxo-ETE-PFB internal standard (*m*/*z* 337 → 120), (e) PGE_2_-PFB (*m*/*z* 351 → 271), (f) [^2^H_4_]-PGE_2_-PFB (*m*/*z* 355 → 275), (g) 13,14-dihydro-15-oxo-PGE_2_-PFB (*m*/*z* 351 → 235), (h) [^2^H_4_]-13,14-dihydro-15-oxo-PGE_2_-PFB (*m*/*z* 355 → 239). Reprinted with permission from Ref. [110].

The LoVo cell line was used to assess the formation of 11-oxo-ETE *in vivo*. LoVo cells are human colorectal carcinoma cells and are expressing both COX-2 and 15-PGDH [20]. When the cells were incubated with 11(*R*)-HETE in presence of NAD^+^, the chiral targeted lipidomics profile showed the presence of 11-oxo-ETE, with same LC-MS characteristics as a synthetic standard. LC-MS analysis showed that 11-oxo-ETE was formed in similar amounts to 15-oxo-ETE. 11-oxo-ETE and 15-oxo-ETE share a common product ion at *m/z* 165, since this ion results from the cleavage of the bond between C-9 and C10, so it was necessary that the two oxo-ETEs to be well separated by the chromatographic run (15-oxo-ETE had a retention time of 12.0 min and 11-oxo-ETE had a retention time of 12.8 min) (**Figure 4**). 15-oxo-ETE was also produced (**Figure 4**) but in lower amount, and the 13,14-dihydro-15-oxo-PGE_2_ was an order of magnitude lower than the 11-oxo-ETE.

Similar experiments were performed with the HCA-7 cells, a human colonic adenocarcinoma line [110]. The HCA-7 cells just have trace amounts of 15-PGDH [114,120] even though COX-2 is expressed at high levels. CAY10397, a 15-PGDH inhibitor, was used to examine its effect on oxidized eicosanoid formation. In the LoVo cells, the concentrations of 11-oxo-ETE, 15-oxo-ETE, and 13,14-dihydro-15-oxo-PGE_2_ were drastically reduced. In contrast, HCA-7 cells, which do not express 15-PGDH, showed no decrease in the levels of 15(*S*)-HETE and PGE_2_.

In the LoVo cells, both 11-oxo-ETE and 15-oxo-ETE reached a maximum concentration at approximately 10 min and then decreased to a steady state concentration over 2.5 h. Due to the rapid clearance of the 11-oxo-ETE in the LoVo cells, its metabolic fate was further investigated. It was shown that the 11-oxo-ETE underwent conjugation with GSH forming the 11-oxo-ETE-GSH adduct (11-OEG), similar to the formation of 15-OEG [19].

11-Oxo-ETE, even though it is acyclic, has the same 11-oxo-moiety as the potent inhibitor of human umbilical vein endothelial cell (HUVEC) proliferation, 15d-PGJ_2_. This might account for the finding that 11-oxo-ETE was six times more potent than 15-oxo-ETE and equipotent with 15d-PGJ_2_ at inhibition of HUVEC proliferation. A HUVEC lysate treated with 11(*R*)-HETE did not produce any 11-oxo-ETE. In keeping with this observation, COX-2 was not detectable by Western blot in the HUVEC lysate.

The targeted chiral lipidomics approach has made it possible to unequivocally demonstrate that 11(*R*)-HETE is a substrate for 15-PGDH and that it is converted to 11-oxo-ETE. This finding has provided another role to 15-PGDH beside inactivation of PGs [110] in which the 11(*R*)-HETE-derived 11-oxo-ETE could exhibit a paracrine anti-proliferative effect on endothelial cells. It is noteworthy that 11-oxo-ETE was detected as an endogenously derived lipid in human atherosclerotic plaques over ten years ago, but the biosynthesis and biological activity were not evaluated at that time [121].

## 4. LOX Mediated Metabolism

### 4.1. 5-Lipoxygenases-Mediated Metabolism of Arachidonic Acid in Human Lymphoblastic Cell Line

**Figure 5:**
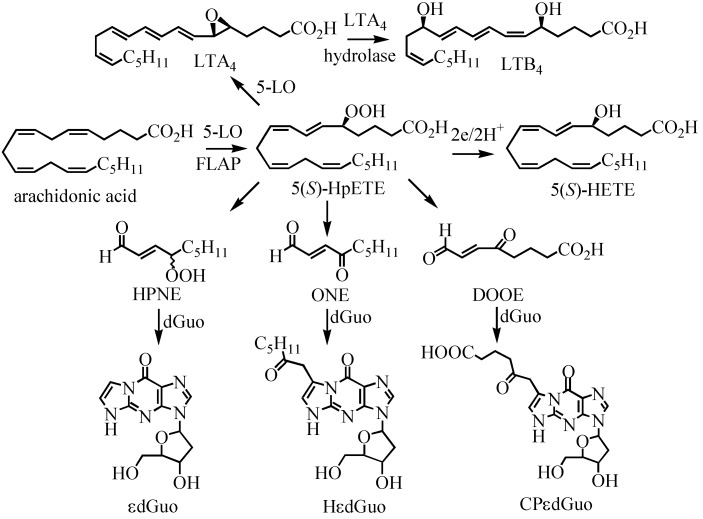
5-LOX-mediated formation of arachidonic acid metabolites and dGuo-adducts. HPNE, 4-hydroperoxy-2(E)-nonenal; DOOE, dioxo-6-octenoic acid. Reprinted with permission from Ref. [108].

5-LOX metabolism is thought to be involved in the etiology of inflammatory diseases [25,122,123]. There are also a number of reports relating inflammation to oxidative stress and cancer. In order to further explore the relationship between oxidative stress and cancer, the CESS cell line, a human lymphoblastoid line, which was established from peripheral blood cells of a patient with myelomonocytic leukemia, was used as model system [40]. Importantly CESS cell express both 5-LOX as well as FLAP. 5-LOX in the presence of FLAP is known to metabolize arachidonic acid to 5(*S*)-HPETE, which is then further reduced to the corresponding 5(*S*)-HETE, or serves as precursor for the formation of LTs (**Figure 5**). Using our targeted chiral lipidomics approach with stable isotope dilution LC-ECAPCI/SRM/MS methodology, the eicosanoid concentrations in this cell line were determined after stimulation with the calcium ionophore A-23187 [40].

**Figure 6:**
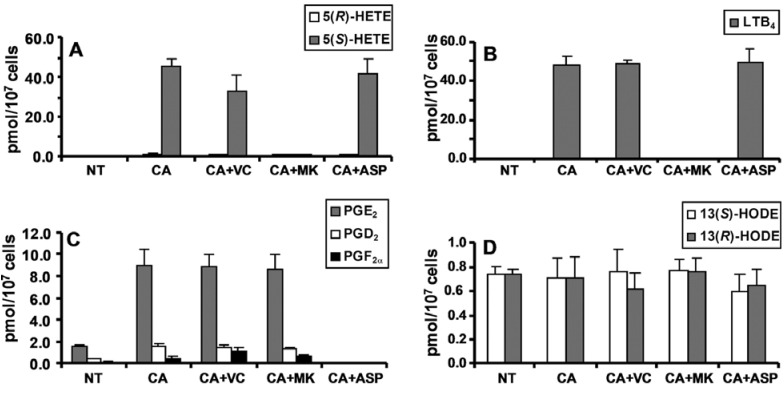
Amount of lipid peroxidation metabolites from CESS cells. A, 5-HETEs. B, LTB4. C, PGE2, PGD2, and PGF2α. D, 13-HODEs. NT, no treatment; CA, treated with 1.0 μm A23187; CA+VC, treated with 1.0 μm A23187 and 1.0 mm vitamin C; CA+MK, treated with 1.0 μm A23187 and 1.0 μm MK886; CA+ASP, treated with 1.0 μm A23187 and 200.0 μm aspirin. Analyses were performed by stable isotope dilution LC-ECAPCI/SRM/MS of PFB derivatives. Determinations were conducted in triplicate (means ± S.D.). Reprinted with permission from Ref. [108].

A targeted lipidomics analysis of the native no treatment (NT) CESS line was conducted after stimulation with the calcium ionophore A-23187. Analyses were also performed after ionophore treatment coupled with inhibition of LOX and COX pathways. 5(*S*)-HETE was used as indirect measurement of 5(*S*)-HPETE formation. Aclear increase in 5(*S*)-HETE formation was observed after treatment with ionophore A-23187 (**Figure 6****A**). When the FLAP inhibitor, MK886 was used together with the calcium ionophore, 5(*S*)-HETE secretion was reduced to levels comparable with the levels observed with the un-stimulated cells. A similar experiment was performed by adding aspirin as inhibitor of COX after treatment with the ionophore. As shown in **Figure 6**, the levels of 5(*S*)-HETE were similar to the calcium ionophore alone, indicating that the 5(*S*)-HETE was largely formed by the 5-LOX pathway. Interestingly, 5(*S*)-HETE concentrations were decreased approximately 25% when vitamin C was added to the media in addition to the ionophore. It is well known that vitamin C is a mediator of lipid hydroperoxide decomposition [124,125]. To further investigate the route of the 5(*S*)-HETE decomposition, a DNA adduct specific for lipid peroxidation was quantified in the same conditions. It was previously shown that *in vitro* reaction of HPETEs with 2’-deoxyguanosine (dGuo) leads to formation of DNA adducts [126,127,128] (**Figure 5**). Two of the DNA adducts [etheno-dGuo (εdGuo) and heptanone-etheno-dGuo (HεdGuo)] were detected in the CESS cells. Interestingly, there was a significant increase in the HεdGuo formation when the CESS cells were treated with vitamin C and the calcium ionophore when compared with the calcium ionophore alone. The amount of the HεdGuo was dramatically decreased if the LOX pathway was inhibited by MK886. The addition of aspirin (a non-specific COX inhibitor) to the CESS cells activated with calcium ionophore had no effect on HεdGuo adduct levels. In contrast, in epithelial cells that stably express COX, the addition of aspirin reduced the HεdGuo levels to basal levels [118]. These studies provided convincing evidence that HεdGuo arose from a LOX- rather than a COX-mediated pathway.

The formation of LTB_4_ by the CESS cells followed a similar pattern to the formation of 5(*S*)-HETE after calcium ionophore treatment (**Figure 6**). However, the addition of vitamin C did not reduce the levels of the LTB_4_. This supported the hypothesis that vitamin C was a inducing the decomposition of the lipid hydroperoxides. PGE_2_, PGD_2_, and PGF_2α_ were the major lipid peroxidation products derived from COX-1-mediated arachidonic acid metabolism. Their levels were increased by the calcium ionophore and were not affected by vitamin C or the MK866 inhibitor (**Figure 6**). All three PGs were reduced to levels lower than the NT level when aspirin was added together with calcium ionophore. Therefore, the targeted chiral lipidomics method was useful for the analysis of enantioselective pathways of cellular LOX and COX mediated arachidonic acid oxidation, being able to differentiate from the racemic mixture formed by a ROS mediated pathway. Additional data provided clear evidence that DNA damage was a result of 5-LOX-mediated arachidonic acid metabolism.

### 4.2. 15-LOX Mediated Metabolism of Arachidonic Acid in Macrophage Cells

**Figure 7:**
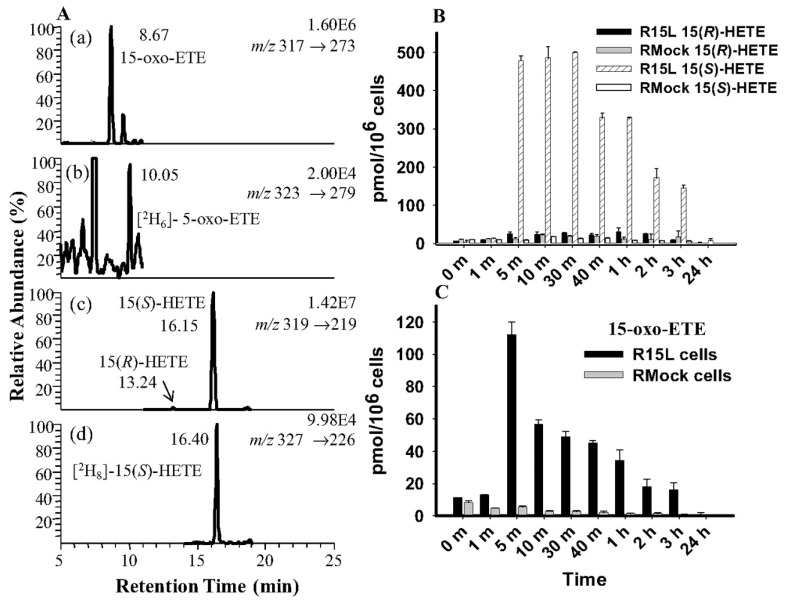
LC-SRM/MS analysis and quantitation of 15-LOX-derived eicosanoids from R15L cells and RMock cells treated with arachidonic acid. A, representative chromatograms of 15-LOX-derived lipid metabolites released by R15L cells after 5-min treatment with 10 μM arachidonic acid. SRM chromatograms are shown for 15-oxo-ETE-PFB (*m*/*z* 317 → 273) (a), [^2^H_6_]5-oxo-ETE-PFB internal standard (*m*/*z* 323 → 279) (b), 15-(*R,S*)-HETE-PFB (*m*/*z* 319 → 219) (c), and [^2^H_8_]15-(*S*)-HETE-PFB internal standard (*m*/*z* 327 → 226) (d). B, concentration-time graph of 15-HETE (*R*- and *S*-form) released by R15L or RMock cells treated with 10 μM arachidonic acid for 24 h. C, concentration-time graph of 15-oxo-ETE released by R15L or RMock cells treated with 10 μM arachidonic acid for 24 h. Cell supernatants were collected at each time point. Lipid metabolites in the cell supernatants were extracted and derivatized with PFB. Determinations were conducted in triplicate (means ± S.E.M.) by stable isotope dilution chiral LC-ECAPCI/SRM/MS analyses of PFB derivatives. Reprinted with permission from Ref [108].

As noted above, the 15-LOXs can metabolize both free and esterified fatty acids [41]. Using a targeted chiral lipidomics approach, 15-LOX products were analyzed in a macrophage cell line. Murine macrophage cells were first transfected with a DNA plasmid containing the human 15-LOX-1 gene, generating the R15L cells. The same line was transfected with an empty plasmid, to generate a control cell line, RMock cells. To examine the 15-LOX-1 activity, the cells were treated with arachidonic acid for 24 h. A chiral LC-MS analysis showed that 15(*S*)-HETE was the main product in the R15L cells followed by 15-oxo-ETE (**Figure 7**). Both eicosanoids reached maximal concentrations after 10 min then declined over 24 h. The level of 15-oxo-ETE was almost 25 % of the level of 15(*S*)-HETE and 15(*R*)-HETE was negligible compared with 15(*S*)-HETE. As expected, in the RMock cells, these three metabolites were close to the detection limit [119]. R15L cells were treated with calcium ionophore to increase the intracellular calcium concentration, which recruits 15-LOX from the cytosol to the inner side of the plasma membrane. The LC-ECAPCI/SRM/MS chromatograms revealed the presence of both 15(*S*)-HETE and 15-oxo-ETE. A time-course analysis showed a maximum concentration at 1 h for 15(*S*)-HETE (18 pmol/10^6^ cells) and at 40 min for 15-oxo-ETE (2 pmol/10^6^ cells). Again, the level of 15(*R*)-HETE was negligible. 

The R15L cells were treated with arachidonic acid or with calcium ionophore, with or without cinnamyl-3,4-dihydroxy-α-cyanocinnamate (CDC; a 15-LOX inhibitor) pre-treatment. CDC was effective in inhibiting the formation of 15(*S*)-HETE by almost 95% in the arachidonic acid-treated cells and of 15-oxo-ETE by almost 70%. CDC almost completely inhibited the calcium ionophore-mediated formation of 15(*S*)-HETE and 15-oxo-ETE. Thus, both 15(*S*)-HETE and 15-oxo-ETE were 15-LOX derived metabolites of endogenous arachidonic acid. To determine the kinetics of the 15(*S*)-HETE metabolism to 15-oxo-ETE, the R15L cells were treated with 15(*S*)-HETE for 3 h. The half-life for the 15(*S*)-HETE was determined to be 21 min, and the peak level for 15-oxo-ETE formation was around 5 min. The half-life for 15-oxo-ETE was 11 min. After 3 h, both metabolites declined to values close to the detection limit. 15-PGDH is responsible for oxidizing the 15(*S*)-hydroxyl group of PGs [129,130]. CAY10397, a selective inhibitor of 15-PGDH was used to determine whether 15-PGDH was the enzyme responsible for transformation of 15(*S*)-HETE to 15-oxo-ETE. The reduction in the levels of 15-oxo-ETE coupled with the accumulation of the15(*S*)-HETE in a dose-dependent manner indicated that indeed 15-PGDH was the enzyme responsible for the conversion of 15(*S*)-HETE to 15-oxo-ETE. The 15-LOX-mediated formation of 15-oxo-ETE has also been observed in human mast cells [131]. 15-oxo-ETE was rapidly cleared from the R15L cells, with a half-life of only 11 min, indicating that it underwent further metabolism. We showed previously that 15-oxo-ETE forms a GSH-adduct through GST-mediated Michael addition [19]. Other studies have shown that arachidonic acid-derived metabolites, such as LTC_4_ and 5-oxo-ETE, can also form GSH-adducts [132,133,134]. This suggests that 15-oxo-ETE was also metabolized to a GSH-adduct in the R15L cells, which would account for its rapid clearance. 

We showed that 15-PGDH-derived 15-oxo-ETE caused inhibition of HUVEC proliferation. It is interesting to note that 15-PGDH is down-regulated in colorectal cancer [22,114]. Therefore, we have speculated that down-regulation of 15-PGDH inhibits the production of 15-oxo-ETE and suppresses the anti-proliferative effect of 15-oxo-ETE on endothelial cells (ECs), thus potentially exacerbating colorectal cancer. Moreover, the capability of 15-oxo-ETE to inhibit EC proliferation suggests that it might be involved in other conditions in which macrophage and/or endothelial cell dysfunction play a role such as in chronic inflammation, atherosclerosis, leukemia, and asthma. Interestingly, 15-LOX-2 is up-regulated in renal tumor infiltrating macrophages [54] suggesting the 15-oxo-ETE could act as a mediator of renal tumorigenesis. Chronic inflammation is known to be involved as a critical component in angiogenesis as well as cancer [135]. Therefore, depending on the location and the local environment *in vivo*, reduction of EC proliferation and migration in response to 15-oxo-ETE treatment might also be responsible for anti-inflammatory activity. Previous studies have demonstrated that over-expression of 15-LOX-1 is associated with an anti-inflammatory response in both rabbit and murine models [136]. Furthermore, aspirin-triggered 15-LOX-1 metabolites of arachidonic acid (LXs) have an anti-inflammatory activity through inhibition of EC proliferation [135,137]. LXs have also been shown to promote resolution, a process known to involve active biochemical programs that enables inflamed tissues to return to homeostasis [137]. 15-LOX-1 activation during the process of inflammation has also been correlated with switching the metabolism of arachidonic acid and other ω-3 polyunsaturated fatty acids to produce pro-resolving lipid mediators such as resolvins and protectins. Taken together, 15-LOX-1 up-regulation can result in the production of anti-inflammatory as well as pro-resolving activities [137]. 

**Figure 8:**
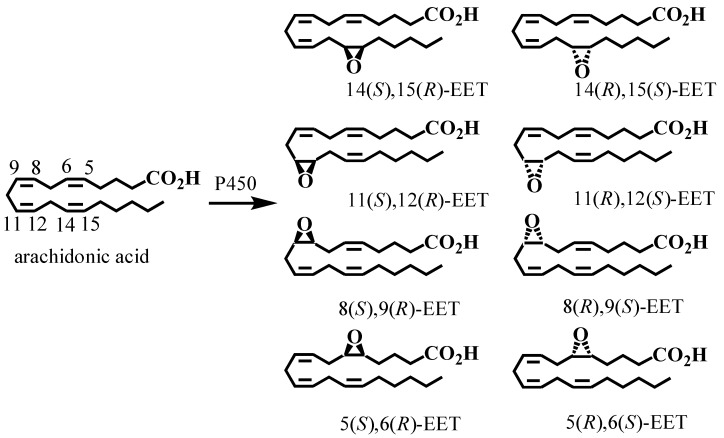
Biosynthesis of epoxyeicosatrienoic acids (EETs) by CYP isoforms. Reprinted with permission from Ref. [138].

## 5. CYP-Mediated Metabolism of Arachidonic Acid

The third pathway for arachidonic acid metabolism involves epoxidation of each *cis*-olefin to produce four EET regioisomers (5,6-EET, 8,9-EET, 11,12-EET, 14,15-EET) each of which can be formed as an enantiomeric pair (**Figure 8**) [66,67,68] . Determination of the enantioselectivity in the formation of the EETs is important in order to determine the major CYPs that are involved. There are multiple reports on the enantiomeric separation of the EET regioisomers using chiral columns with normal or reversed phase chromatography [80,139,140]. However, it has been extremely difficult to determine the enantioselectivity of EET formation in cell and tissue samples because of the problems in analyzing trace amounts of these potent biologically active substances [80,139,140,141]. Typically, this has required initial preparative chiral HPLC separations followed by the analysis of each of the individual isomers using stable isotope dilution GC-ECNCI-MS [142,143] Previous studies have reported the analysis of EETs by LC-MS [144] but the methods did not employ internal standards for the individual enantiomers [145] and long chromatographic run times were required for the chiral separations [142,146].

**Figure 9:**
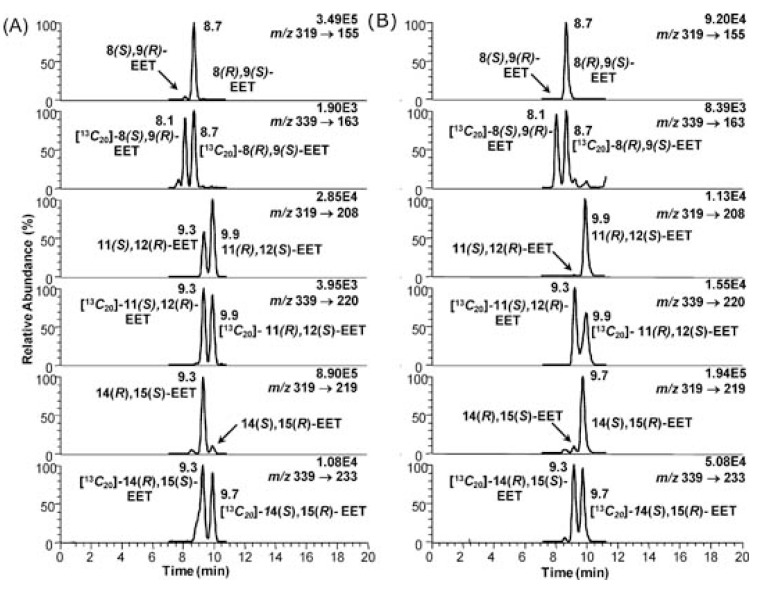
Enantioselective biosynthesis of EETs by CYP family 2 isoforms: (A) hCYP2C19 and (B) hCYP2D6**. **Reprinted with permission from Ref. [138].

EETs have been analyzed using similar LC-ECAPCI/MS methodology to that used for the chiral separation of COX and LOX products. Enantioselectivity of formation from different CYPs isoforms arising from incubation of supersomes with arachidonic acid was then determined (**Figure 9**). Control experiments were conducted in the absence of NADPH in order to assess EET formation that arose from simple autoxidation of arachidonic acid. hCYP2C19 and hCYP2D6 showed unexpected differences in the isomer formation (See Table 1 from ref [138]). 14(*S*),15(*R*)-EET was formed with 96 % enantiomeric excess (ee) by hCYP2D6 but in contrast its enantiomer, 14(*R*),15(*S*)-EET was formed with 96 % ee by hCYP2C19. Both isoforms produced 8(*R*),9(*S*)-EET as almost the only enantiomer, but the enantioselectivity of formation of 11,12-EET was very different, for hCYP2D6 the 11(*R*),12(*S*)-EET was formed almost exclusively (**Figure 9**).

There was a striking difference in the enantioselectivity of 14,15-EET formation between CYP2C19 and CYP2D6 (**Figure 9**). 14(*R*),15(*S*)-EET was formed with a high ee by CYP2C19, whereas 14(*S*),15(*R*)-EET was the predominant enantiomer formed from CYP2D6 (**Figure 9**). As expected, hCYP1A1 and rCYP1A1 had similar enantioselectivity, converting arachidonic acid primarily into the 14(*R*),15(*S*)-EET. CYP-mediated metabolism of arachidonic acid by mouse Hepa cells also resulted in the formation of the EETs with high regioselectivity for 14(*R*),15(*S*)-EET. Hepa cells constitutively express CYP1A1 and CYP1B1, and so a predominance of the 14(*R*),15(*S*)-EET would have been predicted from the supersome studies reported in ref [138]. Up-regulation of these CYPs would also be expected to increase the amounts of EETs that are formed from arachidonic acid.

**Figure 10:**
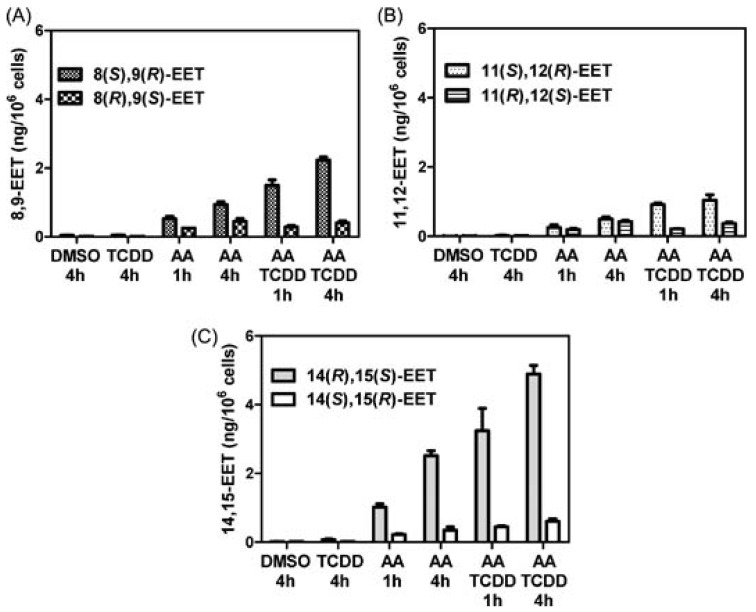
Analysis of epoxyeicosatrienoic acids by chiral liquid chromatography/electron capture atmospheric pressure chemical ionization mass spectrometry using a [^13^C]‐analog internal standards. Reprinted with permission from Ref. [138].

Chiral EET formation in mouse epithelial hepatoma Hepa cells (a rich source of CYPs) was investigated. 2,3,7,8-Tetrachlorodibenzo-p-dioxin (TCDD) is a polychlorinated dibenzo-p-dioxin that binds to the aryl hydrocarbon receptor (AhR), translocates into the nucleus, and up-regulates CYP1A1 and 1B1 expression. The Hepa cells were treated with arachidonic acid with or without TCDD activation. There were two controls, one cells treated with DMSO alone (the vehicle for the inducer) and one cells treated with TCDD only, where no significant levels of EETs were detected. The total amount of EETs (esterified and free) was determined by the same targeted chiral approach. Enantioselective formation of 8(*S*),9(*R*)-EET, 11(*S*),12(*R*)-EET, and 14(*R*),15(*S*)-EET, was observed (**Figure 10**). 14(*R*),15(*S*)-EET was present in the largest amount, followed by 8(*S*),9(*R*)-EET and 11(*S*),12(*R*)-EET (**Figure 10**). The amount of each isomer increased from 1 h to 4 h treatment, in both stimulated and un-stimulated cells. 

After 4 h of arachidonic acid treatment, all the EET regioisomers increased by approximately 50 %, and the enantioselectivity of the EETs was preserved. When the cells were pre-treated with TCDD followed by arachidonic acid, the concentration of all the cellular EETs increased. After adding 10 μM arachidonic acid for 1 h to the TCDD pre-treated cells, the most abundant regioisomer was 14,15-EET (**Figure 10**) and it showed a preferential formation of the 14(*R*),15(*S*)-EET enantiomer. The second most abundant regioisomer was 8,9-EET (**Figure 10**) with the 8(*S*),9(*R*)-EET enantiomer being formed preferentially . Surprisingly, 8(*S*),9(*R*)-EET was the major arachidonic acid-derived 8,9-EET in both the non-induced and TCDD-induced Hepa cells. None of the CYPs that were tested produced significant quantities of this enantiomer, which has been shown previously to be a major metabolite of the rat cortex [78]. This suggests that there is another CYP in the mouse Hepa cell line, which is responsible for the formation of 8(*S*),9(*R*)-EET. Interestingly, the 8(*S*),9(*R*)-EET enantiomer has potent vasoactive proprieties and undergoes COX-mediated metabolism to a potent mitogen for mesangial cells [147,148]. The low abundance of the 8(*R*),9(*S*)-EET in the TCDD-induced cells at 1 h and 4 h, a significant product of both rCYP1A1 and 1B1 suggests that preferential hydrolysis of this EET enantiomer could have occurred as a result of TCDD treatment.

11,12-EET, a minor product of arachidonic acid metabolism of CYP1A1 and 1B1 in the supersomes was also the least abundant product in the Hepa cell incubations. The expected racemic 11,12-EET was observed in the non-induced cells, whereas TCDD induction caused an apparent selective induction of 11(*S*),12(*R*)-EET formation. However, this could have been due to selective hydrolysis of the 11(*R*),12(*S*)-EET isomer as suggested above for 8(*R*),9(*S*)-EET. Taken together, these data suggest that CYP1A1- and 1B1-mediated arachidonic acid metabolism can produce significant quantities of EETs in addition to the widely recognized CYPs of the 2 family. 

## 6. Summary and Future Directions

Targeted chiral LC-SRM/MS analysis of arachidonic acid metabolites has until recently been performed primarily by ECAPCI methodology [105]. This requires derivatization to PFB-derivatives and the use of normal phase chromatography, which has severely restricted its utility. Nevertheless significant progress has been made in monitoring the formation of chiral eicosanoids that result from COX-, LOX-, and CYP-mediated arachidonic acid metabolism. The recent development of chiral reversed-phase methodology promises to make targeted approaches more readily available to other researchers in the field [93]. However, the need for rigorous attention to detail and the requirement for heavy isotope internal standards [149] to ensure specificity, means that it will be difficult to extend these approaches to more global analyses.

The recent identification of the N-arachidonyl-amino acid derivatives of glutamic acid and glutamine [150] as significant metabolites in rat brain and the previous identification N-arachidonyl-glycine, alanine, and dopamine [151,152] will also require the use of chiral LC-MS methodology in order to ensure that no racemization of the relevant amino acids has occurred. Similar methodology will also be required for other polyunsaturated fatty acid derivatives of amino acids such as N-docosahexaenoyl-glutamic acid [150]. As more sophisticated instruments become more widely available, it will be possible to increase specificity of analysis through the use of multiple transitions that are employed in multiple reaction monitoring (MRM) methodology [149]. This will enable several MRM transitions to be employed for qualifying the analyte and another transition to be employed for quantification as we described recently in our serum proteomics studies [153]. Furthermore, new high-resolution LC-SRM/MS methodology is becoming more amenable to high throughput applications. The use of high resolution LC-MS will confer additional much needed specificity for difficult eicosanoid analyses in complex biological fluids such as urine.
